# Concerns regarding “The impact of combining regional nerve block with general anesthesia on cognitive function in patients undergoing elbow joint release surgery: a randomized controlled trial”

**DOI:** 10.1097/JS9.0000000000003008

**Published:** 2025-07-08

**Authors:** Minmin Cai, Lei Shi, Zihan Ma

**Affiliations:** Department of Anesthesiology, the Affiliated People’s Hospital of Ningbo University, Ningbo, Zhejiang, China

*Dear Editor,*

The recent randomized controlled trial published in the *International Journal of Surgery* exploring the impact of combining regional nerve block with general anesthesia on cognitive outcomes in patients undergoing elbow joint release surgery offers meaningful insight into strategies to mitigate postoperative cognitive dysfunction (POCD)[1]. While the findings are of clinical interest, we believe several important considerations merit further discussion. This correspondence is compliant with the TITAN Guidelines 2025—governing declaration and use of AI[2].

First, the reliance on the Mini-Mental State Examination (MMSE) as the primary tool for cognitive assessment, though convenient for broad screening, has notable limitations. The MMSE lacks sensitivity in detecting subtle or specific cognitive deficits, particularly in domains such as executive function, processing speed, and working memory, which are crucial for a patient’s successful return to daily activities[3]. A two-point change in MMSE score, although statistically significant, may not adequately capture nuanced cognitive changes or may be subject to a ceiling effect in patients with higher baseline cognitive function.

Second, while the authors propose several plausible mechanisms, including mitigation of neurotoxic effects, reduction of neuroinflammation, and preservation of cholinergic function, these remain speculative within the current study framework. Neuroinflammation is indeed a leading hypothesis in POCD pathophysiology, driven by pro-inflammatory cytokines triggered during surgical stress and anesthesia[4]. Quantifying inflammatory biomarkers in serum or cerebrospinal fluid (such as C-reactive protein, interleukin-6 and neuron-specific enolase) is essential to provide direct evidence of reduced neuroinflammation[5]. Similarly, assessing markers of cholinergic activity or utilizing advanced neuroimaging techniques to explore cholinergic receptor density would offer stronger empirical support for cholinergic preservation.

Lastly, the acknowledgment of “unmeasured confounders” underscores a key methodological limitation. POCD is multifactorial, and pre-existing mild cognitive impairment, comorbidities (such as diabetes and hypertension), preoperative chronic pain, and postoperative complications including delirium may all exert significant influence on cognitive outcomes[6]. Future studies should include comprehensive baseline neurocognitive profiling and rigorous comorbidity documentation, followed by appropriate statistical adjustment or stratified analysis to mitigate confounding.

## Data Availability

All data are included in the article.
